# Comparative Genomics Revealing the Genomic Characteristics of *Klebsiella variicola* Clinical Isolates in China

**DOI:** 10.3390/tropicalmed9080180

**Published:** 2024-08-16

**Authors:** Fang Yang, Fei-Yi Liu, Yi-Ming Zhong

**Affiliations:** 1 Department of Clinical Laboratory, Xiangya Hospital, Central South University, Changsha 410008, China; 2 National Clinical Research Center for Geriatric Disorders, Xiangya Hospital, Central South University, Changsha 410008, China; 3 Faculty of Laboratory Medicine, Xiangya School of Medicine, Central South University, Changsha 410013, China

**Keywords:** *Klebsiella variicola*, phylogenetic analysis, resistance, virulence genes

## Abstract

*Klebsiella variicola* is an opportunistic pathogen often misidentified as *Klebsiella pneumoniae*, leading to misdiagnoses and inappropriate treatment in clinical settings. The genetic and molecular characteristics of clinically isolated *K. variicola* remain largely unexplored. We aim to fill this knowledge gap by examining the genomic properties of and evolutionary relationships between clinical isolates of *K. variicola*. The genomic data of 70 *K. variicola* strains were analyzed using whole-genome sequencing. A phylogenetic tree was generated based on the gene sequences from these *K. variicola* strains and public databases. Among the *K. variicola* strains, the drug resistance genes with the highest carrying rates were beta-lactamase and aminoglycoside. Locally isolated strains had a higher detection rate for virulence genes than those in public databases, with yersiniabactin genes being the most prevalent. The K locus types and MLST subtypes of the strains exhibited a dispersed distribution, with O3/O3a being the predominant subtype within the O category. In total, 28 isolates carried both IncFIB(K)_Kpn3 and IncFII_pKP91 replicons. This study underscores the importance of developing more effective diagnostic tools and therapeutic strategies for *K. variicola* infections. The continued surveillance and monitoring of *K. variicola* strains is essential for understanding the epidemiology of infections and informing public health strategies.

## 1. Introduction

*Klebsiella variicola* belongs to the Enterobacteriaceae *Klebsiella pneumoniae* species complex (KpSC) and, for years, has been misclassified as *K.pneumoniae*, as they share phenotypic and biochemical characteristics [1]. Studies [2] have shown that approximately 10% of clinical *K. pneumoniae* isolates are actually, for example, *K. Variicola*, *Klebsiella quasipneumoniae*, *Klebsiella aerogenes*, and other subspecies of *Klebsiella*. Like *K. pneumoniae*, *K. variicola* is an opportunistic pathogen that can cause bloodstream, respiratory, and urinary tract infections [3,4,5,6]. Research [3] indicates that *K. variicola* is associated with higher mortality among patients with bloodstream infections than *K. pneumoniae*. In 2019, Farzana et al. [7] reported a significant increase in neonatal mortality due to an outbreak of neonatal sepsis caused by *K. variicola*. Therefore, the clinical importance and significance of *K. variicola* infections are underestimated, and their true prevalence is obscured by imprecise detection methods. Although studies have suggested that *K. variicola* may have a significantly higher virulence, large gaps remain in our understanding of its virulence characteristics, drug resistance traits, and evolutionary processes [1]. Additionally, there are few studies on the genetic and molecular characteristics of clinically isolated *K. variicola*, and data from China have not yet been reported. Therefore, this study aims to reveal the molecular characteristics of *K. variicola* identified as *K. pneumoniae* in clinical practice over six consecutive years in a large comprehensive hospital in China by analyzing genomic data. By comparing these data with *K. variicola* genomic data in public databases, we seek to trace the evolutionary relationships of Chinese clinical isolates of *K. variicola* to provide a basis for the prevention and control of the spread of this bacterium in clinical settings.

## 2. Materials and Methods

### 2.1. Sources of Bacterial Genomic Data

Based on our previous research data [8], strains were selected from 2193 non-repetitive *K. pneumoniae* strains clinically isolated from Xiangya Hospital of Central South University from January 2013 to July 2018. Whole-genome sequencing was performed using the BGISEQ-500 high-throughput sequencing platform (MGI, Shenzhen, China), and we ultimately discovered that 70 strains were *K. variicola*. The whole-genome sequencing results for all strains can be downloaded from the China National GeneBank Database (Accession Number CNP0001198) (https://db.cngb.org/search/project/CNP0001198 (accessed on 8 August 2024)). In addition, we downloaded the genomic data of 335 *K. variicola* strains from the NCBI public database for comparative analysis. This study complied with the ethical principles of human medical research as stated in the Declaration of Helsinki by the World Medical Association, and it was approved by the Medical Ethics Committee of Xiangya Hospital of Central South University (Approval Number: 2020101028).

### 2.2. Genomic Characterization Analysis

To identify the resistance genes, virulence genes, and multilocus sequence typing (MLST) of each isolate, we analyzed the assembled genomes using Kleborate (https://github.com/katholt/Kleborate (accessed on 8 August 2024)), a tool for screening important features of *K. pneumoniae* genome assemblies [9]. The *K. pneumoniae* species complex (KpSC) was assigned sequence types (STs) using previously described methods [10]. Kaptive 2.0 was used to detect K-antigen and O-antigen loci from the assemblies [11]. Resistance genes were detected using nucleotide BLAST alignments against a known allele database, including genes for aminoglycoside (AGly), beta-lactamase (Bla), beta-lactamase inhibitor complex (Bla_inhR), carbapenemase (blaCARB), extended-spectrum beta-lactamase (blaESBL), extended-spectrum beta-lactamase inhibitor complex (blaESBL_inhR), colistin (Col), fosfomycin (Fcyn), fluoroquinolone (Flq), glycopeptide (Gly), macrolide (MLS), phenicol (Phe), rifampicin (Rif), sulfonamide (Sul), tetracycline (Tet), trimethoprim (Tmt), and tigecycline (Tgc). Virulence genes were detected, including yersiniabactin (ybt), colibactin (clb), aerobactin (iuc), salmochelin (iro), rmpADC, and rmpA2. Resistance scores and virulence scores were calculated according to Lam et al. (Table 1) [9]. Plasmid replicon types were identified using the PlasmidFinder version 2.1 database.

### 2.3. Phylogenetic Analysis

We employed kSNP3.0 [12] with a k-mer size of 21 to detect single-nucleotide polymorphisms (SNPs) from concatenated genome sequence data and subsequently constructed maximum likelihood trees. The resulting trees were visualized using FigTree (http://tree.bio.ed.ac.uk/software/figtree/ (accessed on 08 August 2024)).

## 3. Results

### 3.1. Drug Resistance and Virulence Gene Profiles of Klebsiella variicola Isolates from China

**Figure 1:**
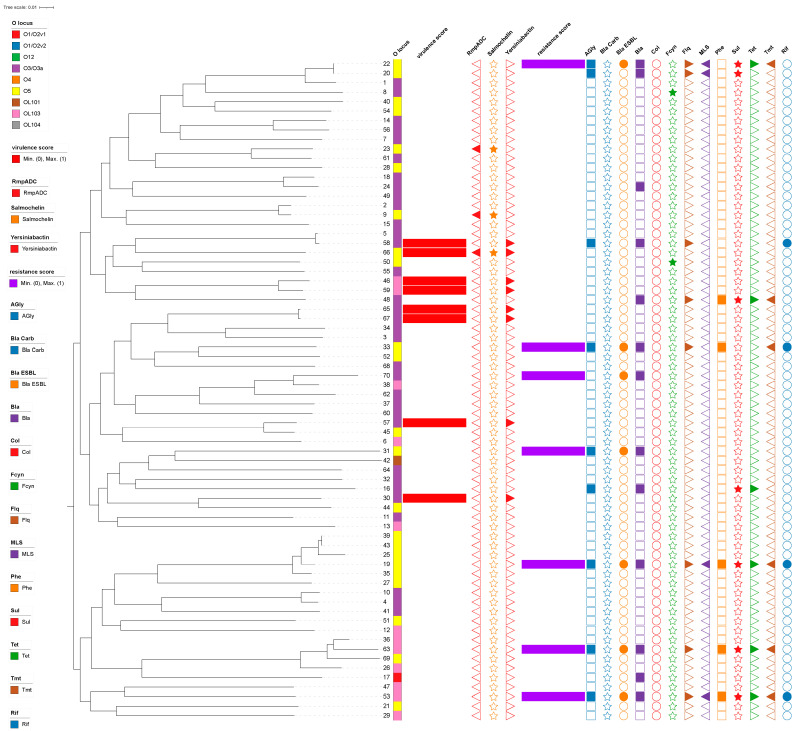
Phylogenetic tree of 70 strains of *K. variicola*. The O locus information is shown as colored strips surrounding the phylogram. Metadata are represented as bars: Resistance and virulence scores in assorted colors are depicted in the legend. Allelic profiling information is shown as colored polygons surrounding the phylogram (from left to right) for RmpADC, Salmochelin, Yersiniabactin, Agly, blaCARB, blaESBL, Bla, Col, Fcyn, Flq, MLS, Phe, Sul, Tet, Tmt, and Rif. Agly: aminoglycoside; blaCARB: carbapenemase; blaESBL: extended-spectrum beta-lactamase; Bla: beta-lactamase; Col: colistin; Fcyn: fosfomycin; Flq: fluoroquinolone; MLS: macrolide; Phe: phenicol; Sul: sulfonamide; Tet: tetracycline; Tmt: trimethoprim; Rif: rifampicin.

Among the 70 *K. variicola* strains, the resistance gene results showed that the highest carrying rate was for Bla, reaching 18.57% (13/70). This was followed by Agly (12.86%, 9/70), blaESBL (10%, 7/70), Fcyn (2.86%, 2/70), Flq (11.43%, 8/70), MLS (5.71%, 4/70), Phe (7.14%, 5/70), Sul (10%, 7/70), Tet (8.57%, 6/70), Tmt (8.57%, 6/70), and Rif (5.71%, 4/70). None of the strains tested positive for blaCARB or Col genes. The virulence gene results indicated that clinical isolates of *K. variicola* carried relatively few virulence genes, the most common being the ybt gene, found in 8/70 (11.43%) of the strains. Next were RmpADC (4.29%, 3/70) and iro (4.29%, 3/70), while no iuc, clb, or rmpA2 virulence genes were detected (Figure 1).

### 3.2. Serotyping and MLST of Klebsiella variicola

The capsule (CPS) covers the surface of bacteria and provides protection. It is a key virulence determinant of *K. variicola* and, in *Klebsiella* spp., typing the K locus helps to identify the capsule types of different strains, which is crucial for understanding the pathogenicity and epidemiological characteristics of these strains. The K locus type (KL) and K type (K) describe the K-antigen typing of *Klebsiella* spp., referred to as K typing. The K type is derived from serological experiments, while the K locus type represents the diversity of the capsule synthesis loci (K-loci). This study identified 41 capsule synthesis loci KL types, including KL16 (n = 4, 5.71%), KL63 (n = 4, 5.71%), KL107 (n = 4, 5.71%), KL34 (n = 3, 4.29%), KL3 (n = 3, 4.29%), KL47 (n = 3, 4.29%), KL57 (n = 3, 4.29%), KL60 (n = 3, 4.29%), KL183 (n = 3, 4.29%), KL113 (n = 2, 2.86%), KL121 (n = 2, 2.86%), KL124 (n = 2, 2.86%), KL135 (n = 2, 2.86%), KL31 (n = 2, 2.86%), KL39 (n = 2, 2.86%), and KL71 (n = 2, 2.86%), among others. The O locus is associated with the bacterial O-antigen, the O-specific chain of lipopolysaccharide. The structural variability of the O-antigen forms the basis for establishing a serotyping scheme. Among the 70 isolated *K. variicola* strains, 47.14% (33/70) belonged to the O3/O3a antigen, 32.86% (23/70) belonged to the O5 antigen, and 17.14% (12/70) belonged to the OL103 antigen; these three antigen types were the main O-antigen types. In the 70 *K. variicola* strains, 57 ST types were detected. The more prevalent ones included ST197 (three strains), ST355 (three strains), and ST360 (three strains), and eight ST types were detected in two strains: ST347, ST357, ST581, ST2362, ST5255, ST5522, ST5479, and ST1096. For details, see Supplementary Table S1.

### 3.3. Plasmid Replicon Typing

We analyzed 65 plasmid replicon types, including ColRNAI, IncFIA, IncFIB, IncFIC, IncFII, IncHI1B, IncHI2, IncHI2A, IncL/M, IncP, IncX, Rep_pKPC-2, and IncR, to determine the importance of plasmid-carried elements in *K. variicola*. We found that most isolates (58/70, 82.86%) carried at least one plasmid replicon type and 47 strains (67.14%) carried multiple plasmid replicons. A total of 23 plasmid replicon types were detected; among them, IncFIB(K)_Kpn3 (n = 40, 57.14%), IncFII_pKP91 (n = 33, 47.14%), ColRNAI (n = 19, 27.14%), IncFII(pHN7A8)_pHN7A8 (n = 12, 17.14%), IncR (n = 8, 11.43%), IncHI1B_pNDM-MAR (n = 7, 10%), and IncFIA(HI1)_HI1 (n = 7, 10%) were the main replicon types. In total, 28 isolates (40.00%) carried both IncFIB(K)_Kpn3 and IncFII_pKP91 replicons, and 10 isolates (14.29%) carried three plasmid replicons: IncFIB(K)_Kpn3, IncFII_pKP91, and ColRNAI. Three strains carried seven plasmid replicons, all of which had ColRNAI, IncFIB(K)_Kpn3, and IncFII(pHN7A8)_pHN7A8 plasmid replicons. The specific plasmid replicon carriage rates are shown in Table 2.

### 3.4. Phylogenetic Analysis of Klebsiella variicola

**Figure 2:**
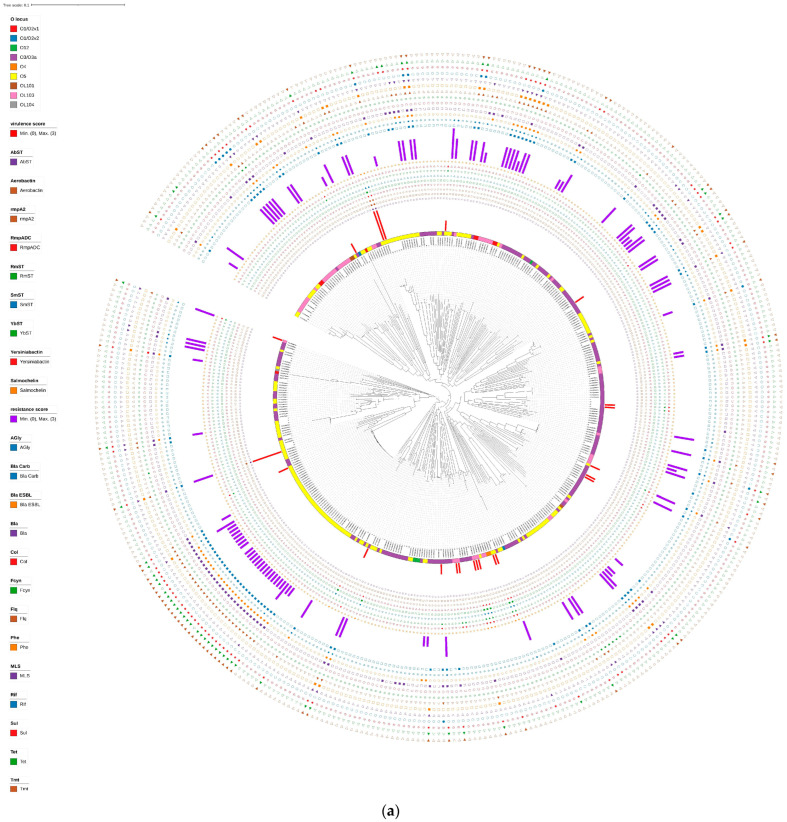

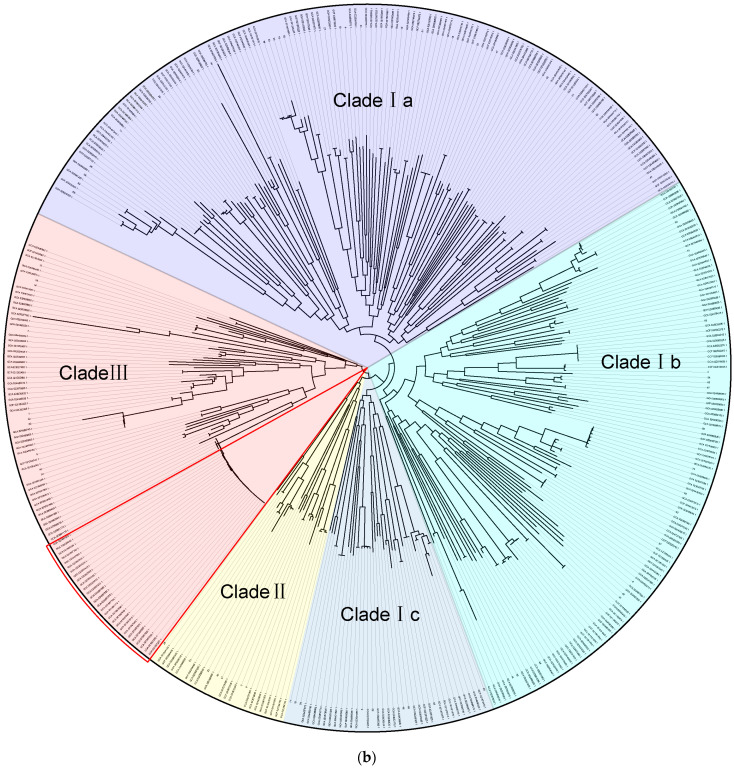
Phylogenetic analysis of 405 *K. variicola* strains (70 local isolate strains and 335 *K. variicola* strains downloaded from public databases). (**a**) Circles outside the tree, from inside to outside, indicate O locus, virulence score, AbST, Aerobactin, rmpA2, RmpADC, RmST, SmST, YbST, Yersiniabactin, Salmochelin, resistance score, Agly, blaCARB, blaESBL, Bla, Col, Fcyn, Flq, Phe, MLS, Rif, Sul, Tet, and Tmt in assorted colors, as depicted in the legend (**b**) Within the circles, bluish violet represents Clade Ia, bluish green represents Clade Ib, blue represents Clade Ic, yellow represents Clade II, pinkish orange represents Clade III, and the red sector frame represents a branch of strains (28 strains) that almost all had a resistance score. Agly: aminoglycoside; blaCARB: carbapenemase; blaESBL: extended-spectrum beta-lactamase; Bla: beta-lactamase; Col: colistin; Fcyn: fosfomycin; Flq: fluoroquinolone; Phe: phenicol; MLS: macrolide; Rif: rifampicin; Sul: sulfonamide; Tet: tetracycline; Tmt: trimethoprim.

A phylogenetic tree analysis (Figure 1 and Figure 2) revealed that the 70 *K. variicola* strains from this region and 335 globally isolated strains, totaling 405 *K. variicola* strains, were mainly divided into three clades. Clade I had the most isolates (293, with 53 from this region), Clade II had 27 (seven from this region), and Clade III had 85 (10 from this region). The Clade I lineage could be further divided into three sub-branches, with clade Ia having 138 strains (26 from this region), Clade Ib having 116 strains (18 from this region), and Clade Ic having 39 strains (nine from this region). The 70 local strains were sporadically distributed overall. Notably, Strains 22 and 20 were almost genetically identical but differed in drug resistance. Strains 5 and 58 showed close phylogenetic relationships in the evolutionary tree but differed in virulence and drug resistance; Strains 46 and 59 and Strains 65 and 67 also had close phylogenetic relationships and possessed certain virulence scores.

The O-antigen loci analysis showed that the main O-antigen types in the *K. variicola* strains downloaded from the public database were O3/O3a (144/335, 42.99%), O5 (133/335, 39.70%), and OL103 (40/335, 11.94%). The O5-type proportion was higher than the strains from our hospital (32.86%), while the O3/O3a-type proportion was lower than our hospital’s strains (47.14%). The virulence scores of the *K. variicola* strains isolated in our hospital were higher than those downloaded from the public database. Among the strains isolated in our hospital, eight strains (8/70, 11.43%) had virulence scores, all of which were 1. In contrast, of the 335 downloaded strains, 14 (14/335, 4.18%) had a virulence score, and three had a score of 3. Eight strains isolated in our hospital tested positive for the ybt virulence locus (11.43%), while the public database detected 11 strains with this locus (11/335, 3.28%). Overall, the detection rate of this locus in our hospital’s strains was higher than in the strains from the public database. In terms of drug resistance, the proportion of strains with a resistance score from our hospital was 11.43%, while the proportion of strains with a resistance score from the public database was 27.04%, much higher than the strains isolated in our hospital. Moreover, the proportion of public strains with a resistance score greater than 3 was also higher than the strains isolated in our hospital. There was a branch of public strains (28 strains) that almost all contained AGly, blaESBL, Bla, Flq, Sul, Tet, and Tmt resistance genes, of which three were not detected with blaESBL, one lacked Flq, two lacked Sul, three lacked Tet, and one lacked Tmt. At the same time, both the strains from our hospital and those downloaded from the public database had a high detection rate for these resistance genes, and the detection rate for the remaining six resistance genes was lower.

## 4. Discussion

*K. variicola* is a novel bacterial species, and strains with hypermucoviscosity and high virulence have been identified, causing high mortality rates in pediatric outbreaks [13,14,15]. In addition, colistin-resistant isolates have emerged, and the chromosomal mechanisms leading to this phenotype have been identified [16]. Although epidemiological and phylogenetic analyses of *K. variicola* have increased, the evolution of its multidrug resistance and virulence gene diversity is still not well-understood [17]. Through comparative *K. variicola* genomics, we found that the co-occurrence of virulence and drug resistance in strains from a particular area in China was rare, and no strains with both high virulence and high drug resistance were found.

This study detected resistance genes in *K. variicola* against multiple drugs and, out of 70 strains, 7 carried ESBL genes, and none carried carbapenem resistance genes. Notably, among the 70 strains, the phylogenetic relationship between Strains 20 and 22 and Strains 5 and 58 was very close. However, Strains 20 and 22 showed differences in drug resistance, while Strains 5 and 58 exhibited differences in drug resistance and virulence. These differences in resistance genes may be due to frequent antibiotics use, leading to mutations in bacterial resistance genes or plasmid transfer. Although no carbapenem-resistant strains were found in this study, a new plasmid-mediated resistance gene cluster encoding tetracycline and tigecycline resistance has emerged in China, and *K. variicola* strains with this plasmid are resistant to tigecycline [18]. If it further spreads to other clinically high-risk *Klebsiella* spp. clones, it may exacerbate the antimicrobial resistance crisis [18]. In the UK, a *K. variicola* strain resistant to IMI-2 carbapenemase has also been found [19]. Therefore, continuously monitoring resistance genes in *K. variicola* is necessary to prevent the spread of these genes.

Previous studies have found that hypervirulent *K. pneumoniae* virulence is associated with virulence genes such as ybt, clb, iuc, iro, and rmpA/rmpA2, which are involved in encoding siderophores, regulators of the mucoid phenotype related to capsules and markers of high virulence [14]. In terms of virulence-related genes, the carriage rate of ybt in *K. variicola* isolates was relatively high (11.43%), followed by iro (4.29%), which was higher than in a study in Japan [20] showing that out of 421 *K. variicola* strains, 1.0% (4/421) were rmpA-positive and 0.2% (1/421) were iro-positive. This study detected virulence sites (ybt, clb, iuc, iro, rmpADC, and rmpA2) according to *K. pneumoniae* subspecies and scored them according to virulence scoring rules. Eight strains detected in our hospital tested positive for yersiniabactin, with a virulence score of 1, but virulence genes such as iuc, clb, and rmpA2 were not detected, which may be related to the epidemiological trends of *K. variicola* in this region or the transfer of virulence plasmids.

The O-antigen loci typing of strains isolated in our hospital mostly showed types O3/O3a and O5. By combining a phylogenetic tree analysis of their kinship, we found that, for example, Strains 2 and 9 and Strains 45 and 57 were genetically very close, yet they had different O-antigens. O-antigen diversity is crucial for bacteria’s interaction with their environment; it constitutes the first line of defense against the immune system and phage infections and has been shown to mediate antibiotic resistance [21]. Studies [9,20] have shown that the MLST of hypervirulent KpSC includes ST23, ST65, ST86, and so on. In addition, a study in one country reported that Strains ST11 and ST231 and other types have high virulence and MDR (multidrug resistance) characteristics, raising public health concerns [9,20]. Fortunately, none of these sequence types were detected in the strains analyzed in this study. However, vigilance is still needed for the emergence and spread of convergent antimicrobial-resistant–virulent ST types so that they can be targeted for surveillance and infection control.

In the evolutionary relationship between the 70 strains from our hospital and the 335 with public genomic data, the SNP phylogenetic tree separated the 405 strains into three main clades, with Clade I having the largest branch. These three branches exhibited greater genetic differences. Moreover, we did not find any signs of localized outbreaks. Most of the strains isolated in our hospital were within Clade I, with only seven strains in Clade II and 10 in Clade III. Observing the strains in our hospital, some had close phylogenetic relationships, such as Strains 20 and 22 being very close, 39 and 43 being close, and 65 and 67 being relatively close, indicating the possibility of in-hospital cross-infection. In addition, no AMR (antimicrobial resistance) hypervirulence convergence events were identified in this region. Previous studies have shown that most convergent strains are due to strains already carrying iuc acquiring AMR, and the acquisition of AMR in high-virulence lineages is particularly frequent in East and Southeast Asia [9]. Therefore, using genomic data to identify sources of infection and assist infection control agencies in continuous monitoring activities is beneficial in reducing the burden of *K. variicola* AMR.

Compared with the 335 strains from the public database, the proportion of strains positive for the ybt virulence locus detected in the 70 strains isolated from our hospital was higher. Of these, Strains 46 and 59 and Strains 65 and 67—which have the ybt virulence locus—were very closely related, suggesting that the high ybt virulence locus detection rate in our hospital strains may have been related to in-hospital infections. According to virulence scoring rules, all strains with a virulence score from our hospital had a score of 1, and among the 335 public strains, three had a virulence score of 3, which may be related to the epidemiological trends of these strains. In addition, the proportion of public strains with a resistance score was much higher than that of the 70 strains isolated from our hospital. In addition, no carbapenem resistance was found in the 70 strains from our hospital, whereas there were 52 carbapenem-resistant strains among the 335 public strains. This may be related to antibiotic use in different regions and *K. variicola* epidemiological trends in different areas.

This study has several limitations: First, a major limitation was a lack of drug susceptibility assessment, as the presence of genes does not always appear phenotypically. Second, this was a single-center study conducted in one region with a relatively small sample size, which may not represent the entire country. Thus, a multicenter study with larger sample sizes should assess the correspondence and epidemiological trends of *K. variicola* genes and antibiotic susceptibility. Third, the location of antibiotic resistance genes and virulence determinants and their external environments were not determined; further research is required to clarify the specific transmission details of resistance and virulence genes and to conduct a comprehensive analysis of the flow and evolution of virulence and drug resistance in *K. variicola*.

## 5. Conclusions

In summary, this study is the first to use whole-genome sequencing to screen and study drug resistance genes, virulence determinants, and plasmid replicon typing in *K. variicola*. *K. variicola* strains in this region were sporadically distributed overall, with fewer resistance and virulence genes but higher virulence than strains in a public database. Continuously monitoring the flow and evolution of virulence and drug resistance in *K. variicola* is necessary.

## Figures and Tables

**Table 1:** Criteria for resistance score and virulence score.

Score	Genes		
resistance score	blaESBL	blaCARB	Col
0	−	−	/
1	+	−	+/−
2	+/−	+	-
3	+/−	+	+
virulence score	ybt	clb	iuc
0	−	−	−
1	+	−	−
2	+/−	+	−
3	−	−	+
4	+	−	+
5	+	+	+

blaESBL: extended-spectrum beta-lactamase; blaCARB: beta-lactamase carbapenemase; Col: colistin; ybt: yersiniabactin; clb: colibactin; iuc: aerobactin.

**Table 2:** Plasmid replicon typing of the 70 strains of *Klebsiella variicola*.

Plasmid Replicons	No. of Strains (n = 70)	Carriage Rate (%)
Col(Ye4449)	1	1.43%
ColpVC	2	2.86%
ColRNAI	19	27.14%
IncFIA(HI1)_HI1	7	10.00%
IncFIB(K)_Kpn3	40	57.14%
IncFIB(Mar)_pNDM-Mar	2	2.86%
IncFIB(pENTAS01)_pENTAS01	4	5.71%
IncFIB(pKPHS1)_pKPHS1	3	4.29%
IncFII(K)	1	1.43%
IncFII(pHN7A8)_pHN7A8	12	17.14%
IncFII(pKPX1)	1	1.43%
IncFII(pRSB107)_pRSB107	2	2.86%
IncFII_pKP91	33	47.14%
IncHI1B_pNDM-MAR	7	10.00%
IncHI2	1	1.43%
IncHI2A	1	1.43%
IncL/M	1	1.43%
IncN	1	1.43%
IncQ1	1	1.43%
IncR	8	11.43%
IncU	1	1.43%
IncX2	2	2.86%
pSL483	1	1.43%
IncFIB(K)_Kpn3 + IncFII_pKP91	28	40.00%
IncFIB(K)_Kpn3 + ColRNAI	13	18.57%
IncFII_pKP91 + ColRNAI	11	15.71%

## Data Availability

The data that support the findings of this study are available from the corresponding author (Y.-M.Z.) upon reasonable request.

## Supplementary Materials

The following supporting information can be downloaded at: https://www.mdpi.com/article/10.3390/tropicalmed9080180/s1, Table S1: Serotyping and MLST of 70 *Klebsiella variicola* strains.
