# Supplementary material for: Comparative Genomics Revealing the Genomic Characteristics of Klebsiella variicola Clinical Isolates in China
Source: Trop Med Infect Dis. 2024 Aug 16;9(8):180. doi: 10.3390/tropicalmed9080180 (PMC11359898; doi:10.3390/tropicalmed9080180)
Supplement: Supplementary file 1 [file tropicalmed-09-00180-s001.zip › tropicalmed-3118989-supplementary.pdf]

Table S1. Serotyping and MLST of 70 *Klebsiella variicola* strains.

Strain	K_locus	O_locus	ST
1	KL16	O3/O3a	ST360
2	KL39	O3/O3a	ST357
3	KL34	O3/O3a	ST5296
4	KL107	O3/O3a	ST2362
5	KL16	O3/O3a	ST581
6	KL56	OL103	ST5495
7	KL34	O3/O3a	ST697
8	KL134	O3/O3a	ST5499
9	KL57	O5	ST357
10	KL39	O3/O3a	ST2362
11	KL54	O3/O3a	ST877
12	KL184	OL103	ST771
13	KL15	OL103	ST5483
14	KL21	O3/O3a	ST3125
15	KL34	O3/O3a	ST1023
16	KL49	O3/O3a	ST1056
17	KL2	O1/O2v1	ST5487
18	KL57	O3/O3a	ST5488
19	KL27	O5	ST355
20	KL60	O5	ST360
21	KL121	O5	ST4348
22	KL60	O5	ST360
23	KL16	O5	ST3950
24	KL47	O3/O3a	ST5264
25	KL125	O5	ST355
26	KL135	OL103	ST347
27	KL10	O5	ST3954
28	KL31	O5	ST3924
29	KL151	OL103	ST4185
30	KL120	O3/O3a	ST5467
31	KL46	O5	ST5266
32	KL58	O3/O3a	ST5267
33	KL60	O5	ST5474
34	KL113	O3/O3a	ST768
35	KL71	O5	ST5271
36	KL113	OL103	ST197
37	KL53	O3/O3a	ST3144
38	KL135	OL103	ST388
39	KL63	O5	ST5255
40	KL124	O5	ST5506
41	KL35	O3/O3a	ST5312

42	KL107	OL101	ST5256
43	KL63	O5	ST5255
44	KL131	O5	ST1582
45	KL121	O5	ST5479
46	KL183	OL103	ST5522
47	KL183	OL103	ST5252
48	KL47	O3/O3a	ST4364
49	KL3	O3/O3a	ST4314
50	KL63	O5	ST2051
51	KL124	O5	ST5279
52	KL71	O5	ST641
53	KL55	OL103	ST355
54	KL158	O5	ST5314
55	KL18	O3/O3a	ST3903
56	KL64	O3/O3a	ST1984
57	KL103	O3/O3a	ST5479
58	KL16	O3/O3a	ST581
59	KL183	OL103	ST5522
60	KL57	O3/O3a	ST5520
61	KL3	O3/O3a	ST906
62	KL3	O3/O3a	ST1181
63	KL128	OL103	ST197
64	KL31	O3/O3a	ST5302
65	KL107	O3/O3a	ST1096-1LV
66	KL63	O5	ST347
67	KL107	O3/O3a	ST1096
68	KL47	O3/O3a	ST5515
69	KL123	O5	ST197
70	KL114	O3/O3a	ST1562
